# Global epidemiology of carbapenem-resistant *Pseudomonas aeruginosa*

**DOI:** 10.1093/jacamr/dlag208

**Published:** 2026-09-17

**Authors:** Lee S Gottesdiener, Michael J Satlin

**Affiliations:** Department of Medicine, Division of Infectious Diseases, Weill Cornell Medicine, New York, NY, USA; NewYork-Presbyterian Hospital, Weill Cornell Medical Center, New York, NY, USA; Transplantation-Oncology Infectious Diseases Program, Division of Infectious Diseases, Weill Cornell Medicine, New York, NY, USA; Department of Medicine, Division of Infectious Diseases, Weill Cornell Medicine, New York, NY, USA; NewYork-Presbyterian Hospital, Weill Cornell Medical Center, New York, NY, USA; Transplantation-Oncology Infectious Diseases Program, Division of Infectious Diseases, Weill Cornell Medicine, New York, NY, USA

## Abstract

Carbapenem-resistant *Pseudomonas aeruginosa* (CRPA) is a common pathogen that is responsible for infections that cause high morbidity and mortality worldwide. Carbapenem resistance in *P. aeruginosa* may be mediated by multiple mechanisms, including changes in outer membrane permeability, efflux pumps, or production of carbapenemases. While approximately one-quarter of *P. aeruginosa* isolates are resistant to carbapenems worldwide, the global distribution of CRPA and carbapenemase-producing isolates varies by geographic region. The proportion of *P. aeruginosa* isolates that are resistant to carbapenems is particularly high in certain countries in Latin America, Eastern Europe, Southeast Asia, Africa, and the Middle East. Carbapenemase-producing CRPA isolates are rare in the USA (2%) but are more commonly encountered in other regions, with the metallo-β-lactamase (MBL) Verona integron-encoded MBL (VIM) predominating in many countries. Patients at greatest risk for infections due to CRPA include hospitalized patients, particularly those in intensive care units, residents of long-term acute-care hospitals, immunocompromised patients, and patients with cystic fibrosis. When traditional antipseudomonal agents lack activity against CRPA, novel β-lactam/β-lactamase inhibitor combinations may be effective treatment options, and cefiderocol may retain *in vitro* activity against MBL-producing CRPA. Continued epidemiologic monitoring and development of new antibiotics will be crucial to combatting the global threat of CRPA.

## Introduction

*Pseudomonas aeruginosa* is an important clinical pathogen due to its ability to cause serious infections, particularly among hospitalized and vulnerable patients. It is among the most common causes of healthcare-associated infections (HAIs) and is associated with increased mortality compared with other pathogens.^1,2^  *P. aeruginosa* is also well-known for its ability to develop resistance to antimicrobial agents via a variety of mechanisms.^3^ Carbapenem-resistant *P. aeruginosa* (CRPA) is of particular significance because carbapenems are relied upon for the treatment of resistant Gram-negative infections, and treatment options for infections due to CRPA are limited. In 2024, the World Health Organization designated CRPA as a high priority pathogen.^4^ Infections due to CRPA lead to increased mortality compared with those due to carbapenem-susceptible *P. aeruginosa*.^5,6^ A systematic analysis of global data from 2019 estimated that there are over 200 000 annual deaths associated with and over 38 000 annual deaths attributable to infections caused by CRPA.^7^ However the geographic impact, mechanisms of resistance, and treatment options for this devastating pathogen are not distributed equally, leading to different challenges posed by CRPA in different geographic regions.^8^ This review aims to provide an overview of the epidemiology of CRPA across global regions, the distribution of important resistance mechanisms, global patterns of susceptibility to antimicrobial agents, and the patients who are most impacted by this pathogen.

The definition of CRPA employed by the US Centers for Disease Control and Prevention (CDC) is resistance to meropenem, imipenem, or doripenem based on Clinical and Laboratory Standards Institute (CLSI) breakpoints [minimum inhibitory concentration (MIC)  ≥ 8 µg/mL].^9–11^ However, many studies use a definition of CRPA that includes carbapenem non-susceptible isolates (MIC ≥4 µg/mL). Moreover, the European Committee on Antimicrobial Susceptibility Testing (EUCAST) has meropenem (MIC ≥16 µg/mL) and doripenem (MIC ≥4 µg/mL) resistance breakpoints for *P. aeruginosa* that differ from CLSI’s.^12^ This review will draw on studies describing both carbapenem-resistant and carbapenem-not-susceptible (NS) *P. aeruginosa*. It is also important to recognize other commonly used categorizations of resistance in *P. aeruginosa*. These include multidrug-resistant, defined as non-susceptibility across three of eight antimicrobial categories, and extensively drug-resistant, defined as non-susceptibility to agents in all but two of these categories.^13^ More recently, difficult-to-treat resistance (DTR) *P. aeruginosa* was defined as resistance to all standard β-lactams and fluoroquinolones.^14^ While these classifications are useful, this review will focus on CRPA given the clinical and epidemiological importance of this subset of *P. aeruginosa*.

## Which patient populations are most affected by CRPA?

CRPA most commonly infects patients at greatest risk for infections caused by *P. aeruginosa*, including those with healthcare exposures, particularly hospitalized patients, and those who are immunocompromised. A study of over 5000 healthcare facilities in 2015–17 by the CDC’s National Healthcare Safety Network (NHSN) found that *P. aeruginosa* was the fourth most common cause of HAI overall, the second most common cause of ventilator-associated pneumonia, the third most common cause of catheter-associated urinary tract infection (CAUTI), and the fifth most common cause of surgical site infection.^1^ In contrast, *P. aeruginosa* rarely causes community-acquired pneumonia or urinary tract infection.^15,16^ Among device-related HAIs due to *P. aeruginosa* reported to the NHSN, 21% were carbapenem resistant.^1^ Several US studies have demonstrated that CRPA is more commonly found in the intensive care unit (ICU) compared with non-ICU settings.^17,18^ However, CRPA is also found outside of the ICU, and in two international studies the majority of CRPA isolates were from patients outside of the ICU.^8,19^ Another important healthcare setting for CRPA are long-term acute-care hospitals (LTACHs). The NHSN found that *P. aeruginosa* was a more frequent cause of CAUTI and central line–associated bloodstream infections in LTACHs compared with hospital ward or ICU settings, and LTACH isolates were more likely to be carbapenem resistant.^1^

Immunocompromised patients are frequently infected with CRPA, particularly those with chemotherapy-induced neutropenia. *P. aeruginosa* is the third most common cause of Gram-negative bacteraemia in patients with fever and neutropenia.^20^ Moreover, *P. aeruginosa* bacteraemia causes significant mortality in neutropenic patients, particularly in patients for whom appropriate therapy is delayed.^21,22^ Thus, the emergence of CRPA presents a significant problem because these isolates are often resistant to recommended empirical therapies in neutropenic patients.^23,24^ One study of allogeneic haematopoietic cell transplant (HCT) recipients found that 30-day mortality was 39% for CRPA bacteraemia compared with 4% for carbapenem-susceptible *P. aeruginosa* bacteraemia.^25^ The prevalence of CRPA in patients with haematologic malignancies and HCT recipients varies by region, with studies from Italy, the Asia-Pacific region, and the USA showing that 71%, 38%, and 14% of *P. aeruginosa* bloodstream isolates, respectively, were carbapenem resistant.^20,26,27^ Breakthrough *P. aeruginosa* infections while patients are receiving fluoroquinolone prophylaxis during neutropenia may be associated with a selection for carbapenem resistance.^28^ CRPA are also notable among solid organ transplant recipients, particularly lung transplant recipients, for whom post-transplant *P. aeruginosa* isolates are often highly resistant.^29^ Studies have found carbapenem resistance to be more common in *P. aeruginosa* bloodstream infections occurring in organ transplant recipients compared with patients without a transplant.^30,31^

Patients with cystic fibrosis (CF) are another population vulnerable to *P. aeruginosa* infections, and a considerable proportion of infections in these patients are caused by CRPA.^32–34^ CRPA is also an important pathogen worldwide in the paediatric population. One global surveillance study of isolates from paediatric patients from 2018 to 2022 found rates of meropenem-NS *P. aeruginosa* isolates to be 20%.^35^ A recent meta-analysis of *Pseudomonas* isolates causing neonatal sepsis in Africa found that 43% were carbapenem resistant.^36^

## Which resistance mechanisms predominate among CRPA?

*P. aeruginosa* is renowned for having a variety of mechanisms of antimicrobial resistance, a trait that is exemplified by its multiple mechanisms of carbapenem resistance, including non-enzymatically mediated mechanisms and carbapenemases (Table 1).^3^ The most important non-enzymatic mechanisms that confer carbapenem resistance in *P. aeruginosa* are modifications to outer membrane porins and upregulation or modifications to efflux pumps. These mechanisms are often related to mutations that can occur upon exposure to carbapenems, which may explain why resistance occurs more commonly during carbapenem therapy for *P. aeruginosa* infections than therapy with other β-lactam agents.^37,38^ The porin OprD is the primary portal of entry for carbapenems across the outer membrane of *P. aeruginosa*.^39^ Therefore, either downregulation or mutational loss of function of this porin can lead to reduced carbapenem susceptibility, although overt resistance may require an additional resistance mechanism.^3^ OprD disruption has been identified in as many as 95% of carbapenem-NS *P. aeruginosa* isolates and over 70% of carbapenem-resistant isolates.^40,41^ OprD inactivation has minimal impact on the activity of cephalosporins or piperacillin because these agents traverse the outer membrane through other porins.^42–44^ This is why many CRPA isolates remain susceptible to ceftazidime, cefepime, and piperacillin-tazobactam, whereas this susceptibility phenotype is uncommon in carbapenem-resistant enterobacterales (CRE).^45–47^

**Table 1.** Mechanisms of antimicrobial resistance among CRPA

Mechanism of resistance	Example(s)	Impact on carbapenem resistance	Impact on resistance to other antipseudomonal agents
Non-enzymatic			
Outer membrane porin modifications	OprD	Imipenem and meropenem^a,b^	Minimal
Efflux pump upregulation	MexAB-OprM, MexXY-OprM	Meropenem > imipenem^b^	Some penicillins and cephalosporins, aztreonam, fluoroquinolones
Penicillin-binding protein mutation	PBP3 (*ftsI* gene)	Meropenem > imipenem	Some penicillins and cephalosporins, aztreonam
Enzymatic			
Serine carbapenemase acquisition	KPC, GES-5	All carbapenems	Penicillins, cephalosporins (including ceftolozane-tazobactam), aztreonam
MBL acquisition	VIM, IMP, NDM	All carbapenems	Most β-lactams (except cefiderocol)
AmpC β-lactamase upregulation and mutations	PDC	Imipenem > meropenem^a^ Ω-loop mutation may restore carbapenem susceptibility	Penicillins, cephalosporins, aztreonam Ω-loop mutations may confer resistance to ceftolozane-tazobactam, ceftazidime-avibactam, and cefiderocol

^a^Imipenem resistance frequently occurs with disruption of OprD combined with PDC.

^b^Meropenem resistance frequently occurs with upregulation of efflux pumps combined with OprD disruption.

Efflux pumps are an important non-enzymatic mechanism of meropenem resistance in *P. aeruginosa*, but not imipenem resistance.^48^ Unlike alterations in OprD, this mechanism impacts other classes of antimicrobial agents because these pumps can expel a variety of antimicrobial agents from the bacterial cell.^49^ The efflux pump MexAB-OprM is constitutively active in *P. aeruginosa*. However, expression of this efflux pump can be upregulated due to mutations in regulatory genes (e.g. *mexR*) upon exposure to certain antimicrobial agents, including carbapenems.^50^ This increased expression can contribute to resistance to carbapenems (although not imipenem), other β-lactams, and fluoroquinolones.^48,51,52^  *P. aeruginosa* additionally harbours other inducible efflux pumps, such as MexXY-OprM, that expel meropenem and a variety of antimicrobial agents, including aminoglycosides, and whose overexpression is also associated with downregulation of OprD.^3,48^ An additional non-enzymatic mechanism of carbapenem resistance in *P. aeruginosa* is modification of penicillin-binding protein 3 (PBP3), an important target of antipseudomonal β-lactams that is encoded by *ftsl*.^53,54^ Certain PBP3 mutations may increase meropenem MIC values more than imipenem MIC values, possibly due to a lower baseline binding affinity of imipenem for PBP3 than meropenem in *P. aeruginosa*.^53^

β-Lactamase enzymes are also important resistance mechanisms in CRPA. Some β-lactamases are intrinsic to *P. aeruginosa* [e.g. *Pseudomonas-*derived cephalosporinase (PDC)] and others are horizontally acquired.^3^ Of the horizontally acquired β-lactamases, some hydrolyse carbapenems and are thus considered carbapenemases. Carbapenemases can be further divided into the Classes A and D serine β-lactamases, and the Class B metallo-β-lactamases (MBLs).^55^ Class A carbapenemases found in *P. aeruginosa* include *Klebsiella pneumoniae* carbapenemase (KPC), as well as specific variants in the Guiana extended-spectrum (GES) family of β-lactamases that possess carbapenemase activity, such as GES-5.^8^ These Class A carbapenemases hydrolyse ceftolozane and are not inhibited by tazobactam, leading to ceftolozane-tazobactam resistance.^56^ In contrast, these carbapenemases are inhibited by avibactam, and thus many KPC-producing and GES-5-producing *P. aeruginosa* remain susceptible to ceftazidime-avibactam. Although many *P. aeruginosa* isolates harbour narrow-spectrum Class D oxacillinases (OXAs), OXA-type carbapenemases, which are more prevalent *Acinetobacter baumannii*, are uncommon in *P. aeruginosa*.^8,57^

MBLs are notable carbapenemases because they are not inhibited by new or old β-lactamase inhibitors, and therefore MBL-producing *P. aeruginosa* are typically resistant to ceftolozane-tazobactam, ceftazidime-avibactam and imipenem-relebactam. The MBLs that are most widely distributed globally in *P. aeruginosa* are VIM, followed by imipenemase (IMP) and New Delhi MBL (NDM).^8,58^ Other MBLs, such as Sao Paulo MBL (SPM),^59^ Adelaide imipenemase (AIM),^60^ and Dutch imipenemase (DIM),^61^ are much less common in *P. aeruginosa* and have a narrower geographic distribution. The acquisition of carbapenemases via plasmids and integrons allows these enzymes to be transferable among *P. aeruginosa* strains.^62^ In addition, these transferable elements frequently harbour genes that encode other resistance determinants, such as aminoglycoside-modifying enzymes and 16S rRNA methyltransferases, that confer resistance to aminoglycosides and fluoroquinolones and render CRPA with limited or no treatment options.^63–65^

PDC is an AmpC β-lactamase that is intrinsic to *P. aeruginosa* and does not typically confer frank carbapenem resistance on its own. However, many common PDC variants increase imipenem MIC values,^66^ so that the addition of OprD loss may lead to imipenem resistance.^67^ Overexpression of PDCs may lead to decreased *in vitro* susceptibility to antipseudomonal β-lactams other than carbapenems.^68,69^ PDC overexpression in *P. aeruginosa* is mediated by the transcriptional regulators AmpR and AmpD.^70^ In addition to overexpression, PDC can also develop mutations that change its hydrolytic profile and serve as important mediators of β-lactam resistance. Of particular concern are mutations in the PDC Ω-loop that increase the hydrolysis of cephalosporins, leading to resistance to new treatment options for CRPA, such as ceftazidime/avibactam or ceftolozane/tazobactam.^71–73^ It should be noted that some PDC Ω-loop mutations that emerge on treatment may actually restore susceptibility to carbapenems.^74^

## What is the global geography of CRPA and its carbapenemases?

While CRPA are found worldwide, exact estimations and comparisons of their prevalence across regions are challenging. Surveillance studies may employ different methodologies or use different criteria to classify CRPA (e.g. NS versus resistance to carbapenems), which may impact the prevalence of carbapenemases and the *in vitro* activity of new antimicrobial agents. Furthermore, the frequency of resistance may vary based on the anatomic sites of the isolates analysed and temporal trends.^75–77^ Additionally, not all global regions are equally represented in surveillance studies.

**Figure 1.**
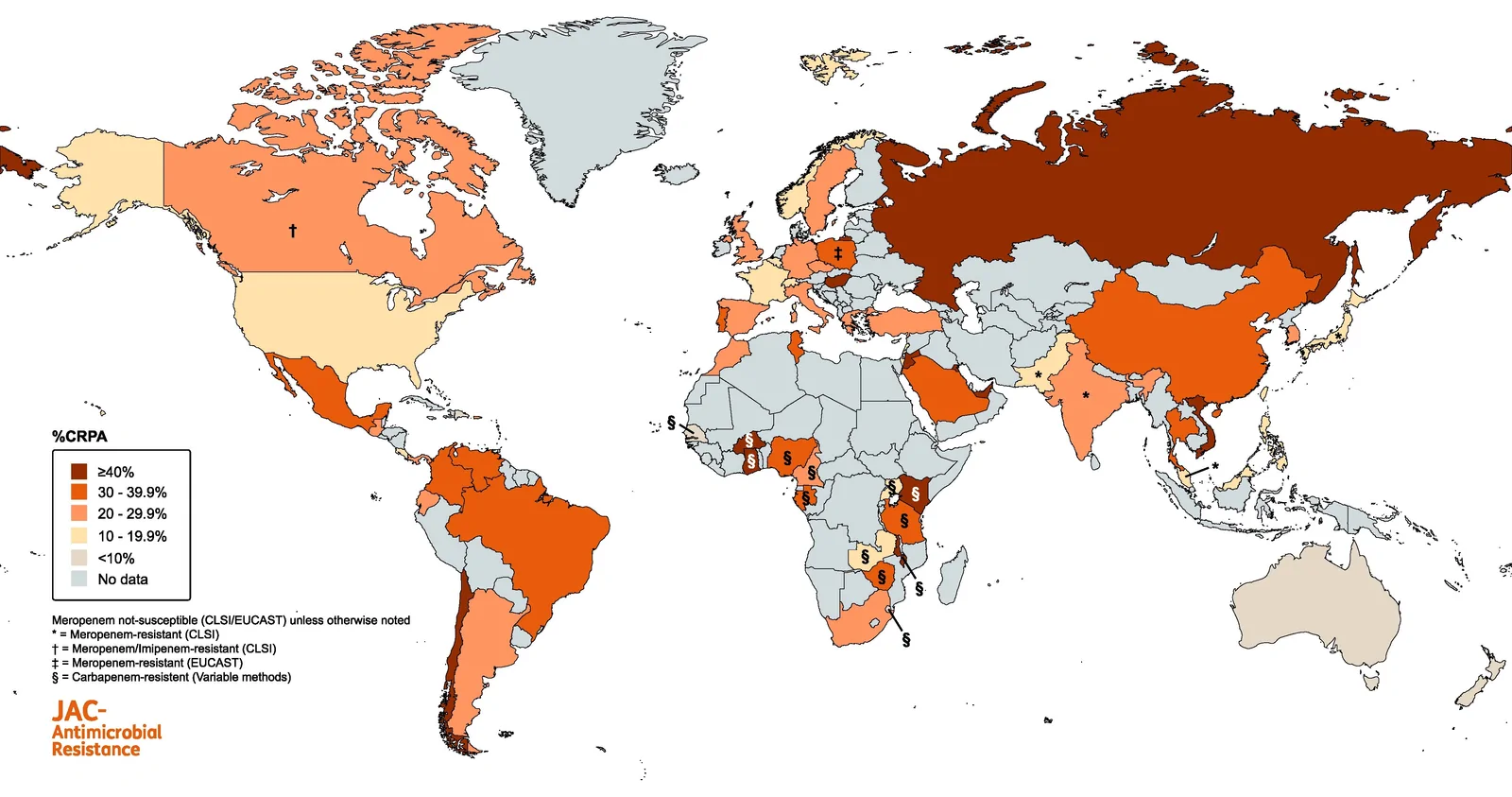
Proportion of *P. aeruginosa* isolates that are carbapenem resistant by country (selected surveillance studies from 2014 to 2023). Surveillance studies selected include programmes led by Antimicrobial Testing Leadership and Surveillance (ATLAS), Canadian Ward Surveillance Study (CANWARD), Mapping Antimicrobial Resistance and Antimicrobial Use Partnership (MAAP), SENTRY, SIDERO-WT, and Study for Monitoring Antimicrobial Resistance Trends (SMART). When multiple studies were available for a given country, studies were selected based on the following criteria: (i) CRPA definition (meropenem resistant prioritized over meropenem-NS); (ii) more recent studies prioritized over older studies; and (iii) number of isolates (studies with <30 isolates for a given country were excluded).^82–97^ Further details for each country and selected references are in Table S1 (available as Supplementary data at *JAC-AMR* Online).

Recent large worldwide surveillance studies conducted between 2018 and 2022 have found that 24%–28% of all *P. aeruginosa* isolates globally were not susceptible to meropenem.^78–80^ These estimates fluctuate over time. A global analysis from 1997 to 2016 found that the rate of meropenem-NS *P. aeruginosa* isolates over 4-year periods ranged from 19% to 27%, peaking during 2009–12.^76^ The global coronavirus disease 2019 pandemic may have also impacted CRPA prevalence. For example, a large study of US veterans found that while the time trend for incident *P. aeruginosa* HAIs had been decreasing in the pre-pandemic period, it subsequently increased from 2020 to 2022.^81^ Regardless of these temporal trends, the greatest degree of variability in the distribution of CRPA is based on geography (Figure 1).^82–97^

The complex geographic distribution of CRPA reflects the non-clonal population structure of *P. aeruginosa*, which is largely composed of heterogenous genotypes.^8,98,99^ This contrasts with the clonal dominance of certain sequence types (STs) of other carbapenem-resistant bacteria, such as ST258 or ST11 in *K. pneumoniae* or ST2 in *A. baumannii*.^46,47,100,101^ However, certain high-risk epidemic clones are important contributors to resistance in *P. aeruginosa*,^98,99^ such as ST235 and ST111, which are associated with a variety of carbapenemases and are found across all global regions.^102,103^

**Figure 2.**
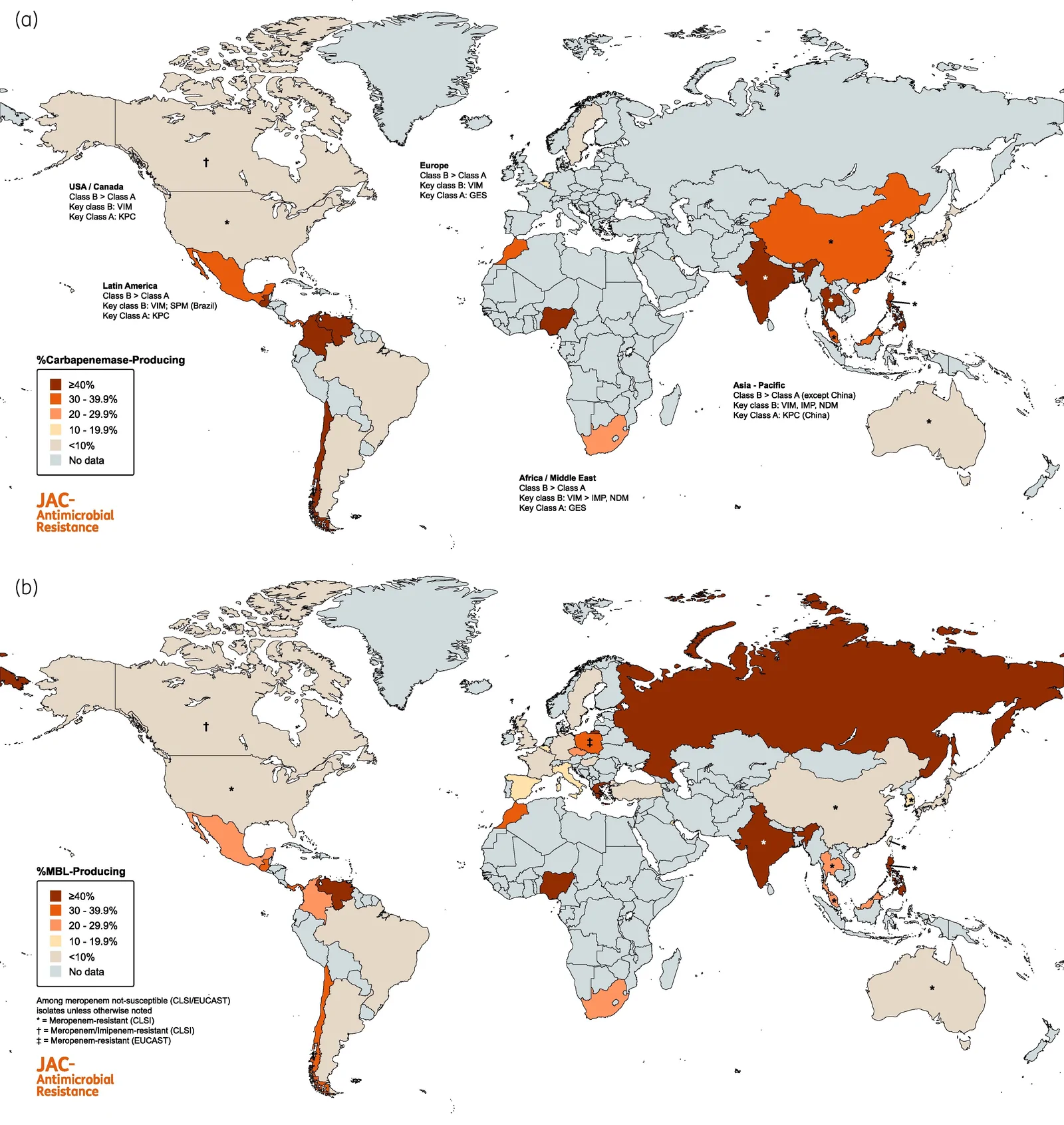
Proportion of CRPA isolates that harbour (a) any carbapenemase and (b) an MBL by country (selected surveillance studies 2014–23). Surveillance studies include programmes lad by Antimicrobial Resistance Leadership Group (ARLG), Antimicrobial Testing Leadership and Surveillance (ATLAS), Canadian Ward Surveillance Study (CANWARD), SIDERO-WT, Study for Monitoring Antimicrobial Resistance Trends (SMART). When multiple studies were available for a given country, studies were selected based on the following criteria: (i) CRPA definition (meropenem resistant prioritized over meropenem-NS); (ii) more recent studies prioritized over older studies; and (iii) number of isolates (studies with <30 isolates for a given country were excluded).^8,83,85,87,88,91,92,104^ Further details for each country and selected references are in Table S2.

There is considerable variability in both the prevalence and types of carbapenemases among CRPA in different geographic regions (Figure 2).^8,83,85,87,88,91,92,104^ Unlike CRE, where the majority of isolates produce carbapenemases across global regions, there are many global regions where carbapenemases are uncommon in CRPA.^8,100,105^ Given that many new antimicrobial agents are targeted towards specific mechanisms of resistance, understanding the distribution of carbapenemases helps predict susceptibilities of CRPA isolates to a variety of antibiotics. Moreover, infections due to carbapenemase-producing CRPA have been associated with increased mortality compared with infections due to CRPA isolates without a carbapenemase.^8^ Additionally, understanding epidemiology may help contain the spread of CRPA and assist clinical microbiology laboratories in deciding on the utility and yield of implementing rapid diagnostic tests to detect carbapenemases in CRPA isolates.

### USA and Canada

In the USA and Canada, surveillance studies suggest a modest prevalence of CRPA compared with other global regions. A surveillance study of 3184 *P. aeruginosa* isolates from US medical centres from 2020 to 2021 found that 18% of isolates were meropenem-NS.^82^ A different surveillance programme of 2524 *P. aeruginosa* isolates from 2019 to 2021 found that 21% of isolates were meropenem-NS.^106^ Within the USA, a survey found that the proportions of meropenem-NS *P. aeruginosa* isolates differed by US regions, with the highest proportion in the Middle-Atlantic (35%) and the lowest in West North Central (16%).^107^ Among CRPA isolates in the USA, the most frequently reported clone is ST235, but this represents only 9% of isolates.^8,10^ The prevalence of CRPA may be similar in Canada to that in the USA. In a study of 1725 *P. aeruginosa* isolates from Canada from 2018 to 2023, 22% were meropenem resistant.^83^

CRPA isolates in the USA have a low rate of carbapenemase production, with the vast majority of carbapenem resistance due to other mechanisms. A CDC study of 54 490 CRPA isolates (resistant to meropenem, imipenem, or doripenem) from 2018 to 2022 detected carbapenemases in only 2% of isolates.^108^ The same percentage (2%) of isolates with carbapenemases was also found in the USA subset of an international study of CRPA, which was the lowest among the global regions evaluated.^8^ Certain US sub-regions may exhibit higher rates of carbapenemase production among CRPA. One study identified carbapenemases in close to 5% of CRPA isolates in the Southeastern USA.^108^ VIM is by far the most common carbapenemase among US CRPA isolates, followed by KPC.^8,108^ The reason that carbapenemases are rare among US CRPA isolates and whether this will remain the case in the future is not clear. Studies of US CRPA isolates have generally found that OprD modification or deletion and/or increased MexAB-OprM expression are the primary mechanisms of carbapenem resistance.^10^

### Latin America

Latin America has a higher frequency of carbapenem resistance in *P. aeruginosa* isolates than the U.SA or Canada. Surveillance studies from Latin America from 2018 to 2020 reported that 26%–33% of *P. aeruginosa* isolates were meropenem-NS.^78,84,85^ Nations with the highest reported proportions of meropenem-NS isolates in recent surveillance studies include Mexico (38%–42%) and Chile (47%).^85,86^ Brazil has also had a dramatic increase in CRPA, increasing from 17% in 2018 to 56% in 2022 in one study.^109^ The high-risk clones ST235 and ST111 are common among CRPA in Latin America, together comprising nearly one-half of CRPA isolates from this region in one surveillance study.^8^

In Latin America, estimates of carbapenemase production among CRPA isolates range from 31% to 69%.^8,58^ VIM is the most common carbapenemase in *P. aeruginosa* in this region, although one study has also reported high rates of KPC.^8,58,110^ Rates of MBLs among CRPA isolates vary by country. One study found MBLs in 5% and 6% of isolates from Brazil and Argentina, respectively, but in 70% of isolates from Venezuela.^85^ Co-carriage of KPC-2 and VIM-2 has also emerged among ST111 *P. aeruginosa* in this region.^8^ The MBL SPM-1 displays an endemic distribution in Brazil and is associated with ST277, although it has recently decreased in prevalence and is now less common than other MBLs.^111–113^

### Europe

A 2017–19 surveillance study that included 9890 *P. aeruginosa* isolates from 22 European countries found that 20% of isolates were meropenem resistant.^110^ However, the distribution of CRPA in Europe varies among regions. For example, a 2018–19 study found that the proportion of *P. aeruginosa* isolates that were meropenem-NS was 19% in Western Europe but 50% in Eastern Europe.^78^ Another study of Northern and Central European *P. aeruginosa* isolates from 2017 to 2021 reported that 18% of *P. aeruginosa* isolates were meropenem-NS.^87^ One surveillance study of 29 European countries identified carbapenem resistance in 15.9% of blood and cerebrospinal fluid *P. aeruginosa* isolates in 2024 according to EUCAST breakpoints, with significant variability across countries.^114^ Countries with the lowest proportion of carbapenem-resistant isolates were Denmark (1.5%) and Finland (2.4%), while those with the highest were Greece (53.4%) and Slovakia (39.4%). In addition to ST235 and ST111, which are among the most important high-risk clones of CRPA in Europe, ST175 is also prominent among CRPA isolates in several European countries.^102,115–117^

Two global surveillance studies that were conducted in 2017–19 and 2019–21, respectively, found that 22% and 27% of CRPA isolates in Europe harboured a carbapenemase.^58,110^ The most common carbapenemase in these studies was VIM, which was detected in 44% and 73% of carbapenemase-producing isolates, respectively.^58,110^ The proportion of CRPA isolates with a carbapenemase also vary by region and country. One study found that the rate of MBLs among *P. aeruginosa* isolates was significantly higher in Eastern Europe compared with Western Europe.^118^ Another study found that Russia (50%) and Greece (44%) had the highest rates of MBLs among meropenem-NS *P. aeruginosa* isolates from 11 European countries, whereas the UK (2%) and France (5%) had the lowest.^88^ All these carbapenemase-producing isolates harboured VIM. Uncommon MBLs in European CRPA isolates include German imipenemase-1 (GIM-1) in Western Germany and Florence imipenemase (FIM-1) in Florence, Italy.^119–121^ The most common serine carbapenemases reported in Europe are GES enzymes.^58,110,115,122^

### Asia-Pacific

The Asia-Pacific region bears a moderate burden of CRPA but also has significant regional variation. Surveillance studies from 2015 to 2020 in this region identified that 19% of *P. aeruginosa* isolates are meropenem resistant and 20%–25% are meropenem-NS.^92,93,123^ Within this region, carbapenem resistance is most common in Southeast Asia and least common in Oceania. A surveillance study found that 45% of *P. aeruginosa* isolates in Vietnam and 33% in Thailand were meropenem-NS.^93^ In contrast, this study found that 8% of *P. aeruginosa* isolates in Australia and New Zealand were meropenem-NS.^93^ The prevalence of carbapenem resistance in *P. aeruginosa* isolates from Hong Kong, South Korea, Malaysia, Philippines, and Taiwan was between 14% and 28%. The reported proportion of *P. aeruginosa* isolates in China that were meropenem resistant was 19% in one surveillance study and 32% in another.^94,124^

In the Asia-Pacific region, the overall rate of carbapenemases among CRPA isolates is approximately 20%, but also varies within sub-regions.^110^ Resistance in many South and Southeast Asian countries is driven by MBLs, where upwards of 40% of all CRPA isolates produce MBLs.^8,92^ In Vietnam, one surveillance study found that more than half of CRPA isolates produced MBLs.^93^ Unlike in other regions where VIM is typically the most common MBL, many of these countries have an equal or higher prevalence of IMP or NDM.^8,92^ A nationwide surveillance study in Japan found that although few CRPA isolates harboured carbapenemases, the majority of carbapenemase-producing isolates possessed IMP genes.^125^ China has a distinct profile, where 32% of CRPA isolates harbour a carbapenemase, but most of these are KPC and few are MBLs.^8^ In one study, over 90% of CRPA isolates from clonal group 463 in China harboured KPC-2.^8^ Several uncommon MBLs have been reported in *P. aeruginosa* in this region, including AIM-1 in Australia and China, Seoul imipenemase-2 (SIM-2) in China, and DIM-1 in South and Southeast Asia.^126–133^

### Africa and Middle East

Africa and the Middle East have historically been less likely to be included in large surveillance studies, but data have emerged in these regions that indicate a high prevalence of carbapenem resistance in *P. aeruginosa*. One surveillance study of 1289 *P. aeruginosa* isolates from 9 sites in 3 African countries found that 28% were meropenem-NS.^95^ The same study also evaluated 2190 *P. aeruginosa* isolates from 19 sites in 7 Middle Eastern countries and found that 25% were meropenem-NS.^95^ A separate study of 1949 *P. aeruginosa* isolates from 5 Middle Eastern countries and 3 African countries found that 28% were meropenem-NS and 20% were meropenem resistant.^104^ One multinational consortium affiliated with the Africa CDC noted concerning extremes in some countries such as Ghana and Burkina Faso, where the proportion of *P. aeruginosa* isolates that were carbapenem-NS was 83% and 68%, respectively.^97^ In the Middle East, some countries such as Jordan, Kuwait and the United Arab Emirates have a >40% reported prevalence of meropenem non-susceptibility among *P. aeruginosa* isolates.^95^ This contrasts with Israel, which has reported rates of only 15% or 18% meropenem non-susceptibility among these isolates.^95,134^

In Africa, rates of carbapenemases among CRPA isolates may be among the highest in the world, with some studies reporting that >60% of these isolates harbour a carbapenemase.^58,95^ The most common carbapenemase reported in CRPA isolates from Africa is VIM, followed by NDM and GES.^58,95,104^ Some countries may have a higher abundance of GES than others, such as Tunisia, where it was found to be more prevalent than MBLs among *P. aeruginosa* isolates.^95^ While data characterizing CRPA isolates from Egypt are limited, there are multiple reports of *P. aeruginosa* isolates producing MBLs that are generally geographically restricted, including SPM, SIM, and GIM.^135–137^

In the Middle East, regional rates of carbapenemases in CRPA isolates have been estimated at 25%–43%.^8,58,95^ Notably, Israel has a lower rate of carbapenemase production than other countries in the region, with a reported rate of < 2%.^95,104^ Among carbapenemases identified in CRPA isolates in the Middle East, VIM is the most common.^8,58,95^

## Which antimicrobial agents have *in vitro* activity against CRPA isolates globally?

The *in vitro* activity of antipseudomonal agents against CRPA isolates varies throughout global regions (Table 2).^56,58,82,84,85,87,92–95,104,106,123,124,138,139^ Some antimicrobial classes that have previously been utilized for CRPA, such as polymyxins and aminoglycosides, have an unfavourable toxicity profile and may have limited efficacy despite their reported *in vitro* activity. Multiple observational studies have found worse outcomes with polymyxin-based regimens for *P. aeruginosa* infections compared with ceftolozane-tazobactam and ceftazidime-avibactam.^140–144^ The CLSI has removed susceptible breakpoints for *P. aeruginosa* for polymyxins, gentamicin, and amikacin (except for urinary isolates for the latter) because of low probability of achieving drug exposure targets that correlate with efficacy using recommended dosages.^11,145,146^ EUCAST has a warning associated with the inadequacy of colistin, tobramycin, and amikacin vs. *P. aeruginosa* isolates and has removed gentamicin breakpoints.^12,145^ Therefore, the antipseudomonal agents for which the global distribution of susceptibilities is most important are β-lactams and fluoroquinolones (Table 2).^56,58,82,84,85,87,92–95,104,106,123,124,138,139^ Unsurprisingly, the distribution of susceptibilities of CRPA isolates globally is influenced by resistance determinants harboured by these organisms in different regions.

**Table 2.** Antimicrobial susceptibilities of CRPA isolates (from multiple anatomic sites) by global region (and sub-regions) published by selected surveillance programmes

Region (sub-region)	Programme	Years	CRPA definition	# of CRPA isolates	% Susceptible							
					C/T	CZA	I/R	P/T	FEP	CAZ	LVX	CIP
USA												
	ARLG^56^	2018–19	Meropenem-R (CLSI)	526	83	77	37	—	—	—	—	—
	ERACE-PA^58^	2019–21	Local CRPA determination	149	85	87	—	—	60	56	—	—
	SMART^106^	2019–21	Meropenem-NS (CLSI/EUCAST)	520	89	80	56	41	49	51	28	—
	SENTRY^82^	2020–21	Meropenem-NS (CLSI/EUCAST)	559	90	85	86	42	52	55	30	48
Latin America												
	SMART^138^	2016–24	Meropenem-R (CLSI)	2715	53	—	—	20	29	30	17	—
	ATLAS^85^	2017–19	Meropenem-NS (CLSI/EUCAST)	835	—	62	—	29	33	35	22	—
	ARLG^56^	2018–19	Meropenem-R (CLSI)	127	34	45	22	—	—	—	—	—
	SMART^84^	2018–20	Meropenem-NS (CLSI/EUCAST)	1433	—	—	41	32	39	40	25	—
	ERACE-PA^58^	2019–21	Local CRPA determination	109	66	75	—	—	50	51	—	—
Europe												
	ERACE-PA^58^	2019–21	Local CRPA determination	324	65	79	—	—	46	52	—	
(Central Europe)	ATLAS^139^	2019	Meropenem-R (EUCAST)	125	—	66	—	18	32	24	9	18
(North/Central Europe)	SMART^87^	2017–21	Meropenem-R (EUCAST)	54	56	—	43	4	15	11	24	—
Asia-Pacific												
	ATLAS^92^	2015–19	Meropenem-R (CLSI)	1198	54	60	—	22	31	33	19	—
	SENTRY^123^	2016–18	Meropenem-NS (CLSI/EUCAST)	94	66	—	—	47	46	48	21	—
	SMART^93^	2017–20	Meropenem-NS (CLSI/EUCAST)	1283	—	—	52	31	38	38	26	—
(Australia and Singapore)	ARLG^56^	2018–19	Meropenem-R (CLSI)	56	36	30	14	—	—	—	—	—
(China)	SMART^94^	2016–19	Meropenem-R (CLSI)	691	55	—	—	14	27	27	17	—
(China)	CHINET^124^	2019	Meropenem or imipenem-R (CLSI)	228	76	68	—	37	50	43	38	48
Africa and Middle East												
	ATLAS^104^	2018–20	Meropenem-NS (CLSI/EUCAST)	524	—	65	—	33	42	43	28	—
(Africa)	SMART^95^	2017–20	Meropenem-R (EUCAST)	198	27	—	—	6	24	25	4	—
(Africa)	ERACE-PA^58^	2019–21	Local CRPA determination	65	32	34	—	—	14	22	—	—
(Middle East)	SMART^95^	2017–20	Meropenem-R (EUCAST)	275	51	—	—	11	28	26	7	—
(Middle East)	ARLG^56^	2018–19	Meropenem-R (CLSI)	91	57	59	30	—	—	—	—	—
(Middle East)	ERACE-PA^58^	2019–21	Local CRPA determination	163	47	57	—	—	42	33	—	—

Abbreviations: ARLG, Antibacterial Resistance Leadership Group; ATLAS, Antimicrobial Testing Leadership and Surveillance; CHINET, China Antimicrobial Surveillance Network; CIP, ciprofloxacin; C/T, ceftolozane-tazobactam; CAZ, ceftazidime; CZA, ceftazidime-avibactam; ERACE-PA, Enhancing Rational Antimicrobials against Carbapenem-resistant *P. aeruginosa*; FEP, cefepime; I/R, imipenem-relebactam; LVX, levofloxacin; MAAP, Mapping Antimicrobial Resistance and Antimicrobial Use Partnership; P/T, piperacillin-tazobactam; R, resistant; SMART, Study for Monitoring Antimicrobial Resistance Trends.

Regions with lower rates of carbapenemases have CRPA isolates that retain a higher proportion of susceptibility to traditional antipseudomonal β-lactams (e.g. ceftazidime, piperacillin-tazobactam). For example, one global study of CRPA isolates from 2019 to 2021 found that in the USA, where <10% of isolates had carbapenemases, 60% of isolates retained susceptibility to cefepime.^58^ In contrast, 46% of CRPA isolates in Europe and only 14% in Africa were susceptible to cefepime, regions where 28% and 63% of isolates harboured a carbapenemase, respectively.^58^ Most surveillance studies of CRPA report slightly lower proportions of isolates that are susceptible to piperacillin-tazobactam compared with antipseudomonal cephalosporins (Table 2), although this may vary by geographic location.^82–89,91–95,104,106,109,124,138,139^ Given that cefepime, ceftazidime, and piperacillin-tazobactam MIC values for *P. aeruginosa* are higher than those for Enterobacterales, optimized dosing strategies are recommended when these agents are used for CRPA infections.^147–150^

Multiple β-lactam agents have become available since 2014 that have expanded our antimicrobial armamentarium against CRPA. At the forefront are the β-lactam/β-lactamase inhibitor (βL/βLI) combinations ceftolozane-tazobactam, ceftazidime-avibactam, and imipenem-relebactam.^151^ Notably, meropenem-vaborbactam does not significantly improve meropenem’s activity against CRPA.^152^ In the USA, susceptibility to the new βL/βLI combinations among CRPA isolates remains high. In studies of USA CRPA isolates from 2018 to 2021, 83%–90% were susceptible to ceftolozane-tazobactam and 77%–87% to ceftazidime-avibactam.^56,58,82,106^ However, reported *in vitro* activity of imipenem-relebactam is more variable, with different studies reporting susceptibilities of 37%, 56%, and 86%, respectively, among CRPA isolates.^52,82,106^ This variability may be related to the inclusion of carbapenem-intermediate isolates in some studies but not in others. Susceptibility to these new βL/βLIs is reduced in regions where more CRPA isolates harbour carbapenemases,^56,58^ particularly MBLs that are not inhibited by any of the βLIs in these combination agents. For example, in a global study of CRPA isolates, 83% of US isolates were susceptible to ceftolozane-tazobactam, compared with only 34% of isolates from South or Central America, where the majority of isolates harboured carbapenemases.^8,56^ The βLIs in these combination agents have varying degrees of inhibitory activity against serine carbapenemases. For example, tazobactam does not inhibit KPC, and neither tazobactam nor relebactam inhibit GES carbapenemases.^152,153^ In contrast, avibactam inhibits both KPC and GES carbapenemases. Thus, in areas where serine carbapenemases are common in CRPA, ceftazidime-avibactam may have greater *in vitro* activity than ceftolozane-tazobactam or imipenem-relebactam.^56,58,152^ For example, in a study of CRPA from Latin America where 32% of isolates harboured KPC as the sole carbapenemase, 45% of isolates were susceptible to ceftazidime-avibactam, 34% to ceftolozane-tazobactam, and 22% to imipenem-relebactam.^8,56^

The siderophore cephalosporin cefiderocol is a novel antimicrobial agent with impressive *in vitro* activity against CRPA. In a global study of 806 CRPA isolates, 98% were susceptible to cefiderocol, whereas only 63%, 72%, and 48% were susceptible to ceftolozane-tazobactam, ceftazidime-avibactam, and imipenem-relebactam, respectively.^154^ Moreover, 96% of MBL-producing CRPA isolates were susceptible to cefiderocol, and these isolates were almost always resistant to the other βL/βLI combinations.^154^ It is important to note that some of these newer agents are not available in many countries, particularly in low- and middle-income countries where the clinical need is greatest.^155,156^ Cefepime-zidebactam was approved by the US Food and Drug Administration in 2026 and has impressive *in vitro* activity against MBL-producing CRPA, but clinical data to support its use for these organisms are lacking.^157,158^

Although fluoroquinolones are an important class of antibiotics for treating *P. aeruginosa*, surveillance studies indicate that only approximately one-quarter of CRPA isolates in the USA and Europe are susceptible to levofloxacin.^87,106^ Other regions, such as the Middle East and Africa, have reported even lower susceptibility of CRPA isolates to levofloxacin.^95^ Some studies have identified slightly higher susceptibility rates of CRPA isolates to ciprofloxacin compared with levofloxacin, although the majority of isolates are still ciprofloxacin resistant.^82,124,139^

### Which antimicrobial agents are recommended to treat CRPA infections?

Appropriate treatment choices for CPRA infections are generally guided by a combination of *in vitro* activity, local availability of antimicrobial agents, and patient and infection characteristics (Table 3). When CRPA isolates test susceptible to traditional antipseudomonal β-lactams (e.g. piperacillin-tazobactam, ceftazidime, cefepime) or fluoroquinolones, these agents are reasonable options.^147,159^ However, in order to achieve drug exposure targets that correlate with efficacy against *P. aeruginosa* infection, these β-lactams should be administered as an extended infusion using the highest dosage.^147,159^ This dosing strategy maximizes the time that the free drug concentration exceeds the MIC value, which is the most important driver of β-lactam efficacy.^160–162^

**Table 3.** Recommended treatments for CRPA infections outside of the urinary tract based on antimicrobial resistance profile

Antimicrobial resistance profile	Suggested antimicrobial agents (IDSA)	Recommended antimicrobial agents (ESCMID)
Carbapenem resistant Susceptible to traditional antipseudomonal β-lactam agents (e.g. piperacillin-tazobactam, ceftazidime, cefepime)	Traditional antipseudomonal β-lactam, using a high-dose, extended-infusion regimen (e.g. cefepime 2 g over 3 hours every 8 hours) Consider novel βL/βLI combinations or cefiderocol if critically ill or ongoing uncontrolled sources of infection	No recommendation
Resistant to traditional antipseudomonal agents (DTR) Susceptible to novel βL/βLI combinations (ceftolozane-tazobactam, ceftazidime-avibactam, imipenem-relebactam)	Ceftolozane-tazobactam, ceftazidime-avibactam, or imipenem-relebactam Ceftolozane-tazobactam preferred for pneumonia	Ceftolozane-tazobactam
Resistant to traditional antipseudomonal agents KPC detected	Ceftazidime-avibactam, imipenem-relebactam, or cefiderocol based on antimicrobial susceptibility testing results	No recommendation
Resistant to traditional antipseudomonal agents and novel βL/βLI combinations (e.g. MBL-producing CRPA)	Cefiderocol Ceftazidime-avibactam plus aztreonam or use of non-β-lactam agents if cefiderocol resistant	Insufficient evidence for cefiderocol If using polymyxins, aminoglycosides, or fosfomycin, use two *in vitro* active agents

When there is *in vitro* resistance to traditional agents, such as in DTR *P. aeruginosa*, novel βL/βLIs or cefiderocol are preferred for isolates with *in vitro* susceptibility to these agents.^14,147,159^ European Society of Clinical Microbiology and Infectious Diseases (ESCMID) guidance favours use of ceftolozane-tazobactam among these options, and the Infectious Diseases Society of America (IDSA) guidance favours ceftolozane-tazobactam for pneumonia, but otherwise prefers this equally with ceftazidime-avibactam or imipenem-relebactam.^147,159^ Preference for one of these agents may be guided by the regional prevalence of carbapenemases among CRPA isolates and regional susceptibilities to these agents.^147^ Given the complexity of β-lactam resistance in *P. aeruginosa* and that most molecular rapid diagnostic assays cannot accurately predict susceptibility to these agents,^3,163^ antimicrobial susceptibility testing should be performed for these newer agents to ensure they are used appropriately. For CRPA that produce KPC, ceftolozane-tazobactam is not recommended, but ceftazidime-avibactam, imipenem-relebactam, or cefiderocol should be considered based on *in vitro* activity.^147^ For CRPA isolates that produce MBLs that confer resistance to these βL/βLIs, cefiderocol is the preferred treatment based on its *in vitro* activity, although clinical data supporting its efficacy for these infections are limited.^164^ Unlike with MBL-producing CRE, aztreonam-avibactam is not recommended for MBL-producing CRPA due to the presence of additional mechanisms of resistance that impact the activity of aztreonam.^147,165,166^ While the combination of ceftazidime-avibactam plus aztreonam may have more *in vitro* activity against MBL-producing CRPA than aztreonam-avibactam alone, clinical data supporting this combination are limited.^167,168^

Novel βL/βLI combinations and cefiderocol are not available in all countries, which may necessitate the use of older agents. Despite their unfavourable toxicity profiles, aminoglycosides or polymyxins may be needed in this setting due to limited treatment options. Tobramycin and amikacin offer the convenience of single-dose therapy for uncomplicated cystitis and of once-daily therapy for complicated urinary tract infection or pyelonephritis.^147,169–171^ Colistin, but not polymyxin B, is an alternative choice for uncomplicated cystitis because it is converted to its active form in the urinary tract.^147,172^ For other severe CRPA infections, if aminoglycosides and polymyxins are the only available treatment options, ESCMID guidance conditionally suggests combining two *in vitro* active agents, and IDSA suggests tobramycin or polymyxin B in combination with a newer β-lactam as last resort.^147,159^ Outside of these situations, combination therapy is generally not recommended for treatment of CRPA.

### Conclusions

CRPA is an important pathogen affecting vulnerable patients worldwide, particularly patients in the ICU or LTACH setting, immunocompromised patients, and patients with CF. The prevalence of carbapenem resistance in *P. aeruginosa* isolates globally is approximately 20%–25% but varies markedly by geographic region. Beyond the differences in the prevalence of carbapenem resistance in each global region, there is marked variability in the distribution of carbapenemases that has a major impact on the effectiveness of available therapies. Areas with higher rates of carbapenemase-producing CRPA, particularly MBLs, may have limitations in the ability to utilize new βL/βLIs for therapy. It is crucial to continue monitoring the global spread of CRPA and its carbapenemases, as well as to develop new antibiotics that are active against CRPA.

## Supplementary Material

dlag208_Supplementary_Data

## Supplementary data

Tables S1 and S2 are available as Supplementary data at *JAC-AMR* Online.
