# Supplementary material for: Global epidemiology of carbapenem-resistant Pseudomonas aeruginosa
Source: JAC Antimicrob Resist. 2026 Sep 17;8(5):dlag208. doi: 10.1093/jacamr/dlag208 (PMC13583628; doi:10.1093/jacamr/dlag208)
Supplement: Supplementary file 1 [file dlag208_supplementary_data.docx]

**SUPPLEMENTARY MATERIAL**

**Table of Contents	Page**

| Table of Contents page | 1 |
| --- | --- |
| Supplemental Table 1. Studies selected to define the proportion of *Pseudomonas aeruginosa* isolates that are resistant to carbapenems in each country (Figure 1). | 2-5 |
| Supplemental Table 2. Studies selected to define the proportion of CRPA isolates that harbor a carbapenemase (CP) or a metallo-β-lactamase (MBL) in each country (Figure 2) | 6-8 |

**Supplementary Table 1**. Studies selected to define the proportion of *Pseudomonas aeruginosa* isolates that are resistant to carbapenems in each country (Figure 1).

| Country | Program/ Reference | Years | CRPA definition | # of *Pa* isolates | # of CRPA isolates | % of CRPA isolates |
| --- | --- | --- | --- | --- | --- | --- |
| **U.S. & Canada** |  |  |  |  |  |  |
| Canada | CANWARD^83^* | 2018-23 | Meropenem/Imipenem-R (CLSI) | 1725 | 371 | 21.5% |
| United States | SENTRY^82^ | 2020-21 | Meropenem-NS (CLSI/EUCAST) | 3184 | 559 | 17.6% |
| **Latin America** |  |  |  |  |  |  |
| Argentina | SMART^84^ | 2018-20 | Meropenem-NS (CLSI/EUCAST) | 527 | 157 | 29.8% |
| Brazil | SMART^84^ | 2018-20 | Meropenem-NS (CLSI/EUCAST) | 610 | 189 | 31.0% |
| Chile | SMART^84^ | 2018-20 | Meropenem-NS (CLSI/EUCAST) | 322 | 184 | 57.1% |
| Colombia | SMART^84^ | 2018-20 | Meropenem-NS (CLSI/EUCAST) | 535 | 177 | 33.1% |
| Costa Rica | ATLAS^85^ | 2017-19 | Meropenem-NS (CLSI/EUCAST) | 55 | 8 | 14.5% |
| Dominican Republic | ATLAS^85^ | 2017-19 | Meropenem-NS (CLSI/EUCAST) | 64 | 5 | 7.8% |
| Ecuador | SMART^84^ | 2018-20 | Meropenem-NS (CLSI/EUCAST) | 149 | 41 | 27.5% |
| Guatemala | SMART^84^ | 2018-20 | Meropenem-NS (CLSI/EUCAST) | 116 | 30 | 25.9% |
| Mexico | SMART^86^ | 2017-21 | Meropenem-NS (CLSI/EUCAST) | 1068 | 405 | 37.9% |
| Panama | SMART^84^ | 2018-20 | Meropenem-NS (CLSI/EUCAST) | 333 | 69 | 20.7% |
| Puerto Rico | SMART^84^ | 2018-20 | Meropenem-NS (CLSI/EUCAST) | 211 | 58 | 27.5% |
| Venezuela | ATLAS^85^ | 2017-19 | Meropenem-NS (CLSI/EUCAST) | 237 | 77 | 32.5% |
| **Europe** |  |  |  |  |  |  |
| Belgium | SMART^87^ | 2017-21 | Meropenem-NS (CLSI/EUCAST) | 360 | 67 | 18.6% |
| Czech Republic | SIDERO-WT^88^ | 2014-19 | Meropenem-NS (CLSI/EUCAST) | 334 | 100 | 29.9% |
| France | SMART^89^ | 2017-20 | Meropenem-NS (CLSI/EUCAST) | 685 | 98 | 14.3% |
| Germany | SMART^89^ | 2017-20 | Meropenem-NS (CLSI/EUCAST) | 679 | 149 | 21.9% |
| Greece | SMART^90^ | 2017-21 | Meropenem-NS (CLSI/EUCAST) | 183 | 53 | 29.0% |
| Hungary | SIDERO-WT^88^ | 2014-19 | Meropenem-NS (CLSI/EUCAST) | 278 | 122 | 43.9% |
| Italy | SMART^90^ | 2017-21 | Meropenem-NS (CLSI/EUCAST) | 911 | 206 | 22.6% |
| Norway | SMART^87^ | 2017-21 | Meropenem-NS (CLSI/EUCAST) | 42 | 8 | 19.0% |
| Poland | ATLAS^91^ | 2015-19 | Meropenem-R (EUCAST) | 543 | 164 | 30.2% |
| Portugal | SMART^89^ | 2017-20 | Meropenem-NS (CLSI/EUCAST) | 373 | 124 | 33.2% |
| Russia | SIDERO-WT^88^ | 2014-19 | Meropenem-NS (CLSI/EUCAST) | 520 | 212 | 40.8% |
| Spain | SMART^89^ | 2017-20 | Meropenem-NS (CLSI/EUCAST) | 978 | 242 | 24.7% |
| Sweden | SMART^87^ | 2017-21 | Meropenem-NS (CLSI/EUCAST) | 280 | 56 | 20.0% |
| Switzerland | SMART^87^ | 2017-21 | Meropenem-NS (CLSI/EUCAST) | 141 | 20 | 14.2% |
| Turkey | SIDERO-WT^88^ | 2014-19 | Meropenem-NS (CLSI/EUCAST) | 369 | 103 | 27.9% |
| United Kingdom | SMART^89^ | 2017-20 | Meropenem-NS (CLSI/EUCAST) | 661 | 133 | 20.1% |
| **Asia-Pacific** |  |  |  |  |  |  |
| Australia | SMART^93^ | 2017-20 | Meropenem-NS (CLSI/EUCAST) | 1061 | 90 | 8.5% |
| China | SMART^94^ | 2016-19 | Meropenem-NS (CLSI/EUCAST) | 2178 | 860 | 39.5% |
| Hong Kong | SMART^93^ | 2017-20 | Meropenem-NS (CLSI/EUCAST) | 331 | 54 | 16.3% |
| India | ATLAS^92^ | 2015-19 | Meropenem-R (CLSI) | 525 | 154 | 29.3% |
| Japan | ATLAS^92^ | 2015-19 | Meropenem-R (CLSI) | 418 | 49 | 11.7% |
| Malaysia | SMART^93^ | 2017-20 | Meropenem-NS (CLSI/EUCAST) | 715 | 99 | 13.8% |
| New Zealand | SMART^93^ | 2017-20 | Meropenem-NS (CLSI/EUCAST) | 457 | 37 | 8.1% |
| Pakistan | ATLAS^92^ | 2015-19 | Meropenem-R (CLSI) | 56 | 10 | 17.9% |
| Philippines | SMART^93^ | 2017-20 | Meropenem-NS (CLSI/EUCAST) | 334 | 62 | 18.6% |
| Singapore | ATLAS^92^ | 2015-19 | Meropenem-R (CLSI) | 45 | 7 | 15.6% |
| South Korea | SMART^93^ | 2017-20 | Meropenem-NS (CLSI/EUCAST) | 707 | 197 | 27.9% |
| Taiwan | SMART^93^ | 2017-20 | Meropenem-NS (CLSI/EUCAST) | 1570 | 298 | 19.0% |
| Thailand | SMART^93^ | 2017-20 | Meropenem-NS (CLSI/EUCAST) | 796 | 262 | 32.9% |
| Vietnam | SMART^93^ | 2017-20 | Meropenem-NS (CLSI/EUCAST) | 412 | 184 | 44.7% |
| **Africa & Middle East** |  |  |  |  |  |  |
| Burkina Faso | MAAP^97^ | 2018-19 | Carbapenem-NS (Variable methods) | 138 | 94 | 68.1% |
| Cameroon | MAAP^97^ | 2017-19 | Carbapenem-NS (Variable methods) | 399 | 108 | 27.1% |
| Eswatini | MAAP^97^ | 2016-18 | Carbapenem-NS (Variable methods) | 54 | 3 | 5.6% |
| Gabon | MAAP^97^ | 2016-18 | Carbapenem-NS (Variable methods) | 78 | 30 | 38.5% |
| Ghana | MAAP^97^ | 2016-18 | Carbapenem-NS (Variable methods) | 40 | 33 | 82.5% |
| Israel | SMART^95^ | 2017-20 | Meropenem-NS (CLSI/EUCAST) | 1126 | 171 | 15.2% |
| Jordan | SMART^95^ | 2017-20 | Meropenem-NS (CLSI/EUCAST) | 106 | 46 | 43.4% |
| Kenya | MAAP^97^ | 2016-18 | Carbapenem-NS (Variable methods) | 167 | 68 | 40.7% |
| Kuwait | SMART^95^ | 2017-20 | Meropenem-NS (CLSI/EUCAST) | 403 | 172 | 42.7% |
| Lebanon | SMART^95^ | 2017-20 | Meropenem-NS (CLSI/EUCAST) | 151 | 29 | 19.2% |
| Malawi | MAAP^97^ | 2016-18 | Carbapenem-NS (Variable methods) | 62 | 29 | 46.8% |
| Nigeria | MAAP^97^ | 2016-18 | Carbapenem-NS (Variable methods) | 545 | 180 | 33.0% |
| Qatar | SMART^95^ | 2017-20 | Meropenem-NS (CLSI/EUCAST) | 217 | 61 | 28.1% |
| Senegal | MAAP^97^ | 2016-18 | Carbapenem-NS (Variable methods) | 279 | 18 | 6.5% |
| Saudi Arabia | SMART^95^ | 2017-20 | Meropenem-NS (CLSI/EUCAST) | 107 | 38 | 35.5% |
| United Arab Emirates | SMART^95^ | 2017-20 | Meropenem-NS (CLSI/EUCAST) | 80 | 35 | 43.7% |
| Morocco | SMART^96^ | 2018-20 | Meropenem-NS (CLSI/EUCAST) | 321 | 74 | 23.1% |
| South Africa | SMART^95^ | 2017-20 | Meropenem-NS (CLSI/EUCAST) | 621 | 165 | 26.5% |
| Tanzania | MAAP^97^ | 2016-18 | Carbapenem-NS (Variable methods) | 226 | 72 | 31.9% |
| Tunisia | SMART^95^ | 2017-20 | Meropenem-NS (CLSI/EUCAST) | 345 | 63 | 32.5% |
| Uganda | MAAP^97^ | 2016-18 | Carbapenem-NS (Variable methods) | 455 | 80 | 17.6% |
| Zambia | MAAP^97^ | 2016-18 | Carbapenem-NS (Variable methods) | 199 | 20 | 10.1% |
| Zimbabwe | MAAP^97^ | 2016-18 | Carbapenem-NS (Variable methods) | 70 | 25 | 35.7% |

For each country, studies were selected for priority based on: 1) CRPA definition (meropenem-not-susceptible prioritized over meropenem-resistant); 2) more recent studies prioritized over older studies; 3) number of isolates (studies with <30 isolates for a given country were excluded).

*This study was selected over a study that used a meropenem-not-susceptible definition because the listed study had more isolates, was more recent, and the proportion of resistant isolates that was similar to the proportion that were not susceptible in the other study.

Abbreviations: ATLAS, Antimicrobial Testing Leadership and Surveillance; Canadian Ward Surveillance Study (CANWARD); CLSI, Clinical and Laboratory Standards Institute; CRPA, carbapenem-resistant *Pseudomonas aeruginosa*; EUCAST, European Committee on Antimicrobial Susceptibility Testing; NS, not susceptible; MAAP, Mapping Antimicrobial Resistance and Antimicrobial Use Partnership; *Pa*, *Pseudomonas aeruginosa*; R, resistant; SMART, Study for Monitoring Antimicrobial Resistance Trends; U.S., United States

**Supplemental Table 2**. Studies selected to define the proportion of CRPA isolates that harbor a carbapenemase (CP) or a metallo-β-lactamase (MBL) in each country (Figure 2)

| Country | Program/ Reference | Years | CRPA definition | # of CRPA isolates | # of CRPA isolates with a CP (MBL) | % of isolates with a CP | % of isolates with an MBL |
| --- | --- | --- | --- | --- | --- | --- | --- |
| **U.S. & Canada** |  |  |  |  |  |  |  |
| Canada | CANWARD^83^* | 2018-23 | Meropenem/Imipenem-R (CLSI) | 371 | 3 (2) | 0.8% | 0.5% |
| United States | ARLG^8^ | 2018-19 | Meropenem-R (CLSI) | 527 | 10 (7) | 1.9% | 1.3% |
| **Latin America** |  |  |  |  |  |  |  |
| Argentina | ATLAS^85^ | 2017-19 | Meropenem-NS (CLSI/EUCAST) | 85 | 7 (5) | 8.2% | 5.9% |
| Brazil | ATLAS^85^ | 2017-19 | Meropenem-NS (CLSI/EUCAST) | 115 | 11 (6) | 9.6% | 5.2% |
| Chile | ATLAS^85^ | 2017-19 | Meropenem-NS (CLSI/EUCAST) | 120 | 60 (44) | 50.0% | 36.7% |
| Colombia | ATLAS^85^ | 2017-19 | Meropenem-NS (CLSI/EUCAST) | 124 | 56 (30) | 45.2% | 24.2% |
| Guatemala | ATLAS^85^ | 2017-19 | Meropenem-NS (CLSI/EUCAST) | 42 | 18 (16) | 42.9% | 38.1% |
| Mexico | ATLAS^85^ | 2017-19 | Meropenem-NS (CLSI/EUCAST) | 238 | 78 (49) | 32.8% | 20.6% |
| Panama | ATLAS^85^ | 2017-19 | Meropenem-NS (CLSI/EUCAST) | 21 | 8 (8) | 38.1% | 38.1% |
| Venezuela | ATLAS^85^ | 2017-19 | Meropenem-NS (CLSI/EUCAST) | 77 | 54 (54) | 70.1% | 70.1% |
| **Europe** |  |  |  |  |  |  |  |
| Belgium | SMART^87^ | 2017-21 | Meropenem-NS (CLSI/EUCAST) | 67 | 10 (10) | 14.9% | 14.9% |
| Czech Republic | SIDERO-WT^88^ | 2014-19 | Meropenem-NS (CLSI/EUCAST) | 100 | NR (21) | NR | 21.0% |
| France | SIDERO-WT^88^ | 2014-19 | Meropenem-NS (CLSI/EUCAST) | 59 | NR (3) | NR | 5.1% |
| Germany | SIDERO-WT^88^ | 2014-19 | Meropenem-NS (CLSI/EUCAST) | 110 | NR (6) | NR | 5.5% |
| Greece | SIDERO-WT^88^ | 2014-19 | Meropenem-NS (CLSI/EUCAST) | 68 | NR (30) | NR | 44.1% |
| Hungary | SIDERO-WT^88^ | 2014-19 | Meropenem-NS (CLSI/EUCAST) | 122 | NR (9) | NR | 7.4% |
| Italy | SIDERO-WT^88^ | 2014-19 | Meropenem-NS (CLSI/EUCAST) | 118 | NR (14) | NR | 11.9% |
| Poland | ATLAS^91^ | 2015-19 | Meropenem-R (EUCAST) | 98 | NR (32) | NR | 32.7% |
| Russia | SIDERO-WT^88^ | 2014-19 | Meropenem-NS (CLSI/EUCAST) | 212 | NR (106) | NR | 50.0% |
| Spain | SIDERO-WT^88^ | 2014-19 | Meropenem-NS (CLSI/EUCAST) | 123 | NR (21) | NR | 17.1% |
| Sweden | SMART^87^ | 2017-21 | Meropenem-NS (CLSI/EUCAST) | 56 | 0 | 0% | 0% |
| Turkey | SIDERO-WT^88^ | 2014-19 | Meropenem-NS (CLSI/EUCAST) | 103 | NR (10) | NR | 9.7% |
| United Kingdom | SIDERO-WT^88^ | 2014-19 | Meropenem-NS (CLSI/EUCAST) | 52 | NR (1) | NR | 1.9% |
| **Asia-Pacific** |  |  |  |  |  |  |  |
| Australia | ATLAS^92^ | 2015-19 | Meropenem-R (CLSI) | 43 | 2 (1) | 4.7% | 2.3% |
| China | ARLG^8^ | 2018-19 | Meropenem-R (CLSI) | 171 | 54 (12) | 31.6% | 7.0% |
| India | ATLAS^92^ | 2015-19 | Meropenem-R (CLSI) | 154 | 93^†^ (64) | 60.4%^†^ | 41.6% |
| Japan | ATLAS^92^ | 2015-19 | Meropenem-R (CLSI) | 49 | 3 (3) | 6.1% | 6.1% |
| Malaysia | ATLAS^92^ | 2015-19 | Meropenem-R (CLSI) | 60 | 19^†^ (13) | 31.7%^†^ | 21.7% |
| Philippines | ATLAS^92^ | 2015-19 | Meropenem-R (CLSI) | 105 | 44 (44) | 41.9% | 41.9% |
| South Korea | ATLAS^92^ | 2015-19 | Meropenem-R (CLSI) | 102 | 17 (13) | 16.7% | 12.7% |
| Taiwan | ATLAS^92^ | 2015-19 | Meropenem-R (CLSI) | 71 | 1 (0) | 1.4% | 0% |
| Thailand | ATLAS^92^ | 2015-19 | Meropenem-R (CLSI) | 144 | 63^†^ (35) | 43.8%^†^ | 24.3% |
| **Africa & Middle East** |  |  |  |  |  |  |  |
| Israel | ATLAS^104^ | 2018-20 | Meropenem-NS (CLSI/EUCAST) | 53 | 1 (1) | 1.9% | 1.9% |
| Kuwait | ATLAS^104^ | 2018-20 | Meropenem-NS (CLSI/EUCAST) | 89 | 16 (14) | 18.0% | 15.7% |
| Morocco | ATLAS^104^ | 2018-20 | Meropenem-NS (CLSI/EUCAST) | 50 | 17 (16) | 34.0% | 32.0% |
| Nigeria | ATLAS^104^ | 2018-20 | Meropenem-NS (CLSI/EUCAST) | 41 | 28 (26) | 68.3% | 63.4% |
| South Africa | ATLAS^104^ | 2018-20 | Meropenem-NS (CLSI/EUCAST) | 76 | 18 (16) | 23.7% | 21.1% |

For each country, studies were selected for priority based on: 1) CRPA definition (meropenem-not-susceptible prioritized over meropenem-resistant); 2) more recent studies prioritized over older studies; 3) number of isolates (studies with <30 isolates for a given country were excluded).

*This study was selected over a study that used a meropenem-not-susceptible definition because the listed study had more isolates, was more recent, and the proportion of resistant isolates that was similar to the proportion that were not susceptible in the other study.

^†^In the referenced study, Vietnamese Extended-Spectrum (VEB) β-lactamase was included among class A carbapenemases and makes up a portion of the reported number of carbapenemases for these countries.

Abbreviations: ATLAS, Antimicrobial Testing Leadership and Surveillance; Canadian Ward Surveillance Study (CANWARD); CLSI, Clinical and Laboratory Standards Institute; CRPA, carbapenem-resistant *Pseudomonas aeruginosa*; EUCAST, European Committee on Antimicrobial Susceptibility Testing; NS, not susceptible; MAAP, Mapping Antimicrobial Resistance and Antimicrobial Use Partnership; NR, not reported; *Pa*, *Pseudomonas aeruginosa*; R, resistant; SMART, Study for Monitoring Antimicrobial Resistance Trends; U.S., United States
